# A Review of Printable Flexible and Stretchable Tactile Sensors

**DOI:** 10.34133/2019/3018568

**Published:** 2019-11-11

**Authors:** Kirthika Senthil Kumar, Po-Yen Chen, Hongliang Ren

**Affiliations:** ^1^Department of Biomedical Engineering, Medical Mechatronics Laboratory, National University of Singapore, Singapore 117583; ^2^Department of Chemical and Biomolecular Engineering, National University of Singapore, Singapore 117585

## Abstract

Flexible and stretchable tactile sensors that are printable, nonplanar, and dynamically morphing are emerging to enable proprioceptive interactions with the unstructured surrounding environment. Owing to its varied range of applications in the field of wearable electronics, soft robotics, human-machine interaction, and biomedical devices, it is required of these sensors to be flexible and stretchable conforming to the arbitrary surfaces of their stiff counterparts. The challenges in maintaining the fundamental features of these sensors, such as flexibility, sensitivity, repeatability, linearity, and durability, are tackled by the progress in the fabrication techniques and customization of the material properties. This review is aimed at summarizing the recent progress of rapid prototyping of sensors, printable material preparation, required printing properties, flexible and stretchable mechanisms, and promising applications and highlights challenges and opportunities in this research paradigm.

## 1. Introduction

The advancement in additive manufacturing and development of material science has kept up with the innovation in robotics, wearable electronics [1], epidermal electronic systems [2], human-machine interfaces [3], soft robotics [4], other biomedical devices [5, 6], and the related systems [7]. In most of these systems, its sensory feedback plays an important role in contemplating the efficiency and performance accuracy. Hence, a tactile sensor is required to emulate the human perception of touch through parameters that define the contact between the object and the sensor such as pressure [8], strain [9], shear [10], force [11, 12], vibration [13], bend, and torsion. Common transduction principles explored that have proven to potentially be used in this type of sensors are piezoresistive, piezocapacitive, piezoelectric, and triboelectric. The recent progress in the field of tactile sensors is made by improving their mechanical flexibility and stretchability. The fact that these flexible and stretchable devices require configurations that conform to the shape of the object in contact calls for more adaptable fabrication methods that are able to deliver the complex geometries and precisely scribed architectures. Hence, 3D printing or additive manufacturing (AM) which is a layer by layer fabrication process, without the need for machining or molds [14], have gained overwhelming attention in the realization of complex and multifunctional objects such as robotic sensing elements [15–19], wearable sensor technologies [20], and flexible sensors in devices [21, 22].

**Figure 1:**
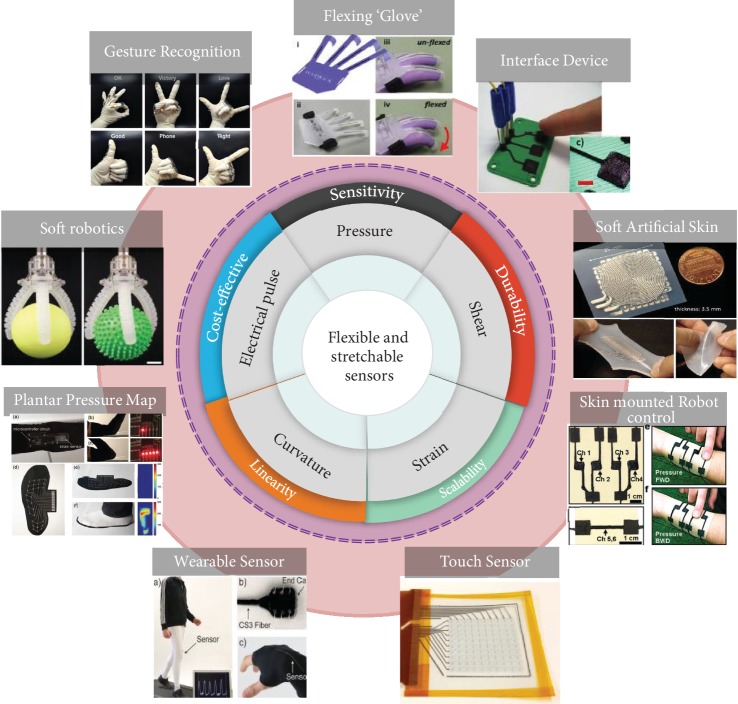
Rapid prototyping methods, their advantages, and the applications of recently developed tactile sensors. “Flexing ‘Glove'” [217], reproduced with permission. Copyright PLOS, 2012. “Interface Device” [217], reproduced with permission. Copyright PLOS, 2012. “Soft Artificial Skin” [218], reproduced with permission. Copyright IEEE, 2012. “Skin mounted Robot control” [219], reproduced with permission. Copyright John Wiley and Sons, 2014. “Touch Sensor” [220], reproduced with permission. Copyright American Chemical Society, 2015. “Wearable Sensor” [39], reproduced with permission. Copyright John Wiley and Sons, 2015. “Plantar Pressure Map” [109], reproduced with permission. Copyright John Wiley and Sons, 2017. “Soft robotics” [40], reproduced with permission. Copyright John Wiley and Sons, 2018. “Gesture Recognition” [105], reproduced with permission. Copyright Royal Society of Chemistry, 2016.

Owing to the advantages this fusion brings to the table, for being time-efficient and easy, attainable complexity, cost-effective manufacturing, and scalability, this combination of rapid prototyping of tactile sensors has invited a lot of attention and interesting applications as shown in Figure 1. Compared to the conventional techniques used to fabricate the tactile sensors, this method is advantageous by firstly avoiding the usage of tools, dies, and molds, in turn, reducing the wear and tear costs. Secondly, it enables the designers to visualize the printed product in a CAD model and make the necessary modifications on the prototype, which allows only using the required materials to build the sensing element which reduces the material wastage. Thirdly, by facilitating the fabrication of intricate design geometries, which would nearly be impossible by conventional fabrication methods, in a single step makes it time and energy-efficient.

**Figure 2:**
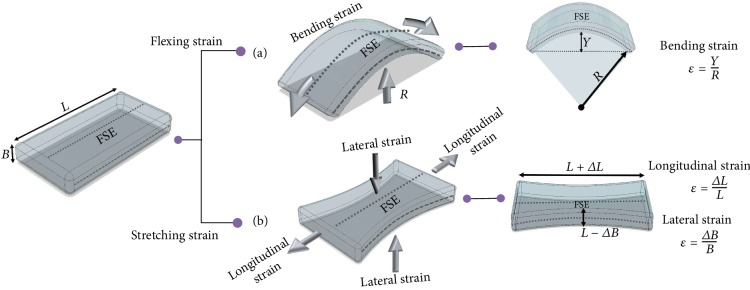
Schematic illustration of flexible (F) and stretchable (S) printed elements. The dotted lines represent the neutral line. (a) Flexibility defined as the bendability and the deflecting ability of the sensor from the plane with a radius of curvature, *R*. *Y* represents the deflection of the sensor from the neutral line. (b) The longitudinal strain caused by stretching increases the length of the printed FSE along the direction of load, while the lateral strain caused by stretching decreases the dimension perpendicular to the direction of load.

Flexible/stretchable tactile sensors fabricated by this manufacturing technique are able to maintain their functional performance in both the original and the deformed states. They typically consist of active elements and a substrate. The substrate is used as the base, for encapsulation or mixed together as composites with active elements. The electrically conductive/active materials widely used are metallic nanoparticles, nanowires, liquid metal, and carbonaceous components like carbon nanotubes, graphene, and other conductive polymers. Throughout this paper, flexibility (F) would refer to the bending and deflection ability of the sensor. Stretchability (S) would refer to the elastic capability of the sensor to accommodate axial and planar strains and resume its former shape and size as explained in Figure 2. This review thus presents an extensive analysis of recent 3D printable flexible/stretchable tactile sensors along with the concerned components such as the various methods of rapid prototyping of sensors, printable ink preparation, requirements of the printers, and functionally suitable materials in terms of properties of the active and substrate materials used. We further discuss the flexibility- and stretchability-enhancing mechanisms through printing, sensing mechanisms of sensors, desired features of the sensors, promising applications, performance, and challenges in this research paradigm.

## 2. Brief Overview of Prototyping Techniques for Flexible/Stretchable Tactile Sensors

Compared to the various fabrication schemes developed to fabricate the tactile sensors, 3D printing has assured to be a promising technology due to its simplicity in system, low cost, scalability, and customization. It has now progressed to be a versatile technology that allows for the production of customized parts through noncontact printing [23–25] while enabling the possibility of flexible/stretchable tactile sensors to be constructed with a significantly lower fabrication cost barrier and scale up the production with desired 3D configurations. There have also been extensive studies on the various 3D printing techniques [26] and its diverse applications [20]. Despite the evident progress in recent years, one of the main challenges in establishing additive manufacturing techniques for large-scale applications is the constricted range of suitable material properties such as mechanical stability, porosity, and anisotropy [27]. These challenges are further explained in the later sections.

**Figure 3:**
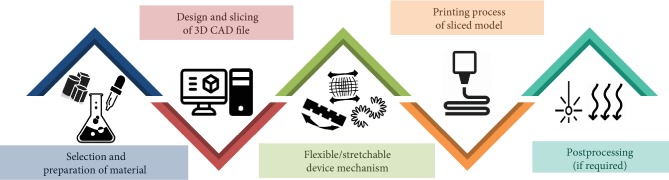
The different phases in the fabrication of printable flexible/stretchable tactile sensors.

In general, rapid prototyping has been ideally used for bringing virtual concepts to physical models. The fabrication of the flexible/stretchable tactile sensors starts with a practical computer-aided design (CAD) model which is then digitally sliced. During the design phase, the alterations that would occur during flexing or stretching of sensors should be taken into account. The selection of the right flexible/stretchable element (FSE) materials and customizing it with the required properties are basic requirements (the following sections), after which the strategies to implement flexibility/stretchability in the sensor devices are to be established. The fundamental printing mechanism for the fabrication for a flexible/stretchable tactile sensor requires a nozzle to extrude or jet the right amount of material. For certain materials, additional UV or laser curing is required for solidification as a postprocessing procedure. All the above-mentioned phases are schematically explained in Figure 3. However, the order is subject to change depending on the type and specifications of the fabricated sensors. The American Society for Testing and Materials (ASTM) has laid out a set of terminologies and a structure for grouping the additive manufacturing techniques [28]. Among those, the fabrication techniques trending in the field of soft, flexible/stretchable tactile sensors with the required features are explained in Table 1 and in the following sections below.

Fused filament fabrication (FFF) is a filament material extrusion-based FSE printing technique. Materials with low melting temperature processed in the form of filaments are commonly used. The nozzle heats up and melts the filaments into a molten or semimolten form which is then deposited in a computer-controlled layer by layer basis on the printer bed. After each iterative slice in a plane, either the printing platform moves down or the printing head moves up. Upon resting on the substrate, the molten material fuses into a solid object. The dependence on the melting and cooling processes restricts the printer usage to thermoplastics and limited polymer composites. Adhesion of the base layer to the platform is an essential step in this AM process. New conductive material composites (poly(ionic liquid)/polymethyl methacrylate (PMMA)/MWCNT composite) are being developed and made into filaments, possessing desired mechanical and electrical properties for the FFF printing of tactile sensors [29]. The nozzle diameter restricts the resolution of the FFF printing. The temperature of the nozzle has been adjusted according to the melting point of the filament material to avoid clogging or to prevent creating voids in the sensors.

Aerosol jet technology is a versatile system that has the ability to handle inks with a wide range of viscosities ranging from 1 to 2500 cP [30]. The FSE printing process aerodynamically focusses the aerosolized microdroplets of material on both planar and nonplanar surfaces by a sheath gas. The aerosolization of the FSE liquid particles (diameter of 20 nm to 5 *μ*m) is done with the help of an atomizer in the system. They are capable of producing a highly focussed and fine beam which could go up to a tenth of the size of its nozzle. They have a printing speed up to 200 mm/s. As the high-resolution patterns could go below 20 *μ*m, a control on the overspray needs to be established [31].

The inkjet printing technique propels the FSE functional ink droplets onto the substrate via a nozzle, producing a fast and accurately patterned film through a thermal resistor or piezoelectric transducer mechanism [32]. The solvent in the deposited FSE fluidic ink droplets evaporates, or the ink components polymerize resulting in a solid. This noncontact type of printing method delivers discrete liquid droplets and reduces material wastage, minimizing contamination and damage to various layers. It caters to the low viscosity FSE inks ranging from 1 to 20 cP, for piezoelectric printheads (5-20 cP) and thermal printheads (1-5 cP) [33]. The typical surface energy of the fluids is kept below 0.1 J/m^2^ [34]. The printed FSE trace is greatly affected by the printing speed of the nozzle. A bimodal sensor that has the ability to determine the bending strain and pressure stimulus was fabricated by inkjet printing. The smallest line spacing and width of the printed features were reported to be 66 and 155 *μ*m, respectively [35]. Due to its high resolution, it has a potential application in the deposition of etching fluids, to selectively etch and produce the desired pattern with a manageable process [36].

**Figure 4:**
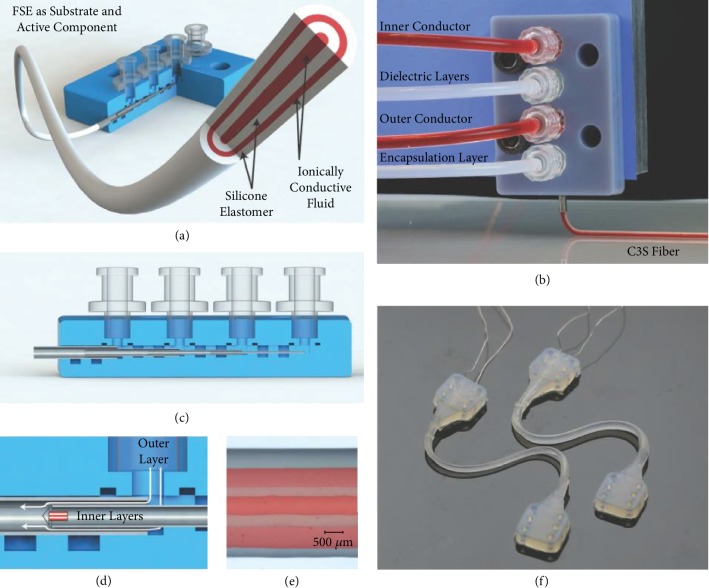
(a) Schematic illustration of the multicore-shell fiber printing process of capacitive strain sensors in a four-layer configuration. (b, c) The conductive and elastomeric inks are loaded into separate reservoirs. (d) Illustrations of the outlet region where the printing simultaneously forms the multicore-shell. (e) Magnified optical image of the printed multicore-shell segmented view. (f) Two fully printed capacitive strain sensors [39]. Reproduced with permission. Copyright John Wiley and Sons, 2015.

**Figure 5:**
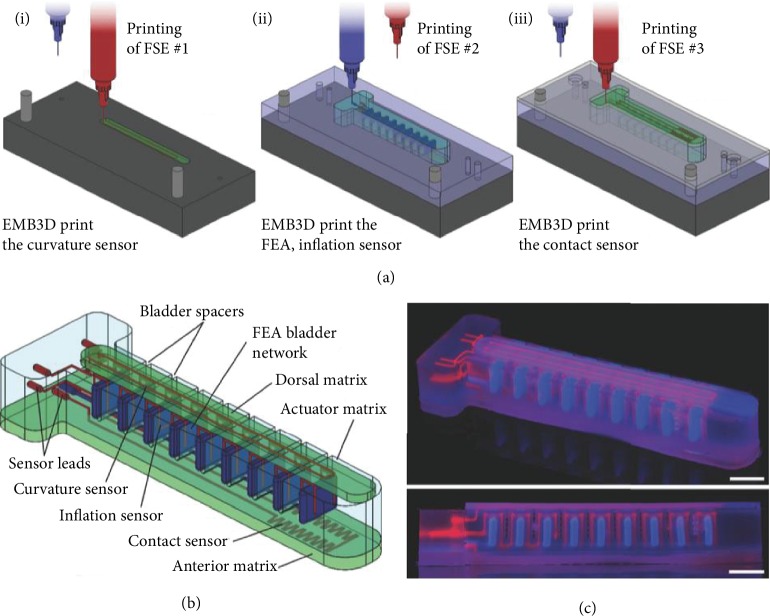
The fabrication steps of the embedded 3D printing of actuators innervated with sensors [40]. Reproduced with permission. Copyright John Wiley and Sons, 2018.

Direct ink writing is considered as the versatile extrusion-based technique and is preferred for FSE materials with a wide range of viscosities up to 10^6^ MPa s. Solidification of the printed FSE material wholly depends on the rheological properties of the functional ink. The ink/paste is extruded from the pneumatic or syringe nozzle with the help of compressed air. Thus, it reduces the possibility of nozzle clogging. It is a common type of technology where custom-built printers are developed with aided properties for printing such as Weissenberg effect, which favors the sensing performance of the sensors [37]. Coaxial direct printing of active material along with the nonconductive elastomeric encapsulant with the core-shell configuration has attracted attention over the years [38]. This produced a concentrically aligned multicore-shell fiber stretchable strain sensor that gave a stable output for strains up to 250%. The four-layered configuration has an overall filament thickness of 1.5 mm, with its inner core diameter as 336 *μ*m, as shown in Figure 4 [39]. Recently, the embedded 3D printing (EMB3D) method gained popularity due to its ability to print multimodal, multimaterial sensors (Figure 5) [40, 41]. Though the direct printing technique is more versatile compared to the other manufacturing processes, there is still room for the development of ink that is able to stabilize itself in the same printed structure. Minimization of fluid flow after printing and rapid solidification of the soft materials is essential.

Electrohydrodynamic (EHD) jet printing technique uses the help of the electric field to polarize the FSE mobile ions in the solution, causing Coulomb repulsion. These ions get accumulated near the surface, deforming the meniscus to a conical shape (Taylor cone). When the electric field builds up to the critical limit, it leads the electric stress to overcome the surface tension, hence emitting a droplet. The resulting feature resolution of the printed FSE trace range is about 240 nm to 5 *μ*m [42]. The critical parameters to consider for this type of printing would be the distance between the nozzle, mobility of ions, and applied voltage [43]. Material jetting refers to the propelling of small droplets of functional ink onto various substrates, such as paper, plastic, and polymers by using a narrow printhead like a nozzle. The film produced using this technique is accurate, uniform, and reproducible. These droplets solidify in response to heat or light. The liquid ink is heated up within the reservoir forming a bubble upon the application of heat. This forces the ink droplet out of the nozzle in the “pull” action [44].

In addition to the above-mentioned techniques, Laser Direct Writing (LDW) is another technique that could potentially be used to pattern the FSE substrate or the other sensor components [45]. This laser-induced deposition uses laser pulses to regulate the structure and properties of the materials. Its resolution ranges from nanometer scaling up to millimeters. However, this method is expensive and requires sophisticated equipment. This process does not allow deposition of FS organic substrates, which omits most of the materials used for the fabrication of flexible/stretchable tactile sensors [46]. It is limited to deposit materials on flat substrates [47].

Due to their assuring qualities of these printing techniques, they aid in the fabrication of the tactile sensors. These fabrication methods are utilized separately or even as a combination [48]. A hybrid 3D printing process for a stretchable tactile sensor device utilized projection stereolithography to build the compliant substrate and the direct printing technology to print the sensing element [49]. A 3D printing process was proposed to activate polyvinylidene fluoride (PVDF) polymer as a piezoresponsive material by integrating it with the corona poling process. In this technique, the poling electric field that promotes the alignment of the dipole moments was created by using the nozzle of the printer as the anode and the heating bed as the cathode [50].

Besides the printing of the sensor materials such as the sensing element, sensor body, or sensing electrode, which is well documented in Table 2, these methods are also used to fabricate sensor molds [51, 52]. Further details on the materials and sensor properties are listed in Table 3 in the future sections. A highly sensitive flexible capacitive sensor was fabricated by creating a 3D printed mold. This mold was microstructure patterned in order to improve the sensor's sensitivity [53]. Silicone and hydrogel layers were created using these 3D printed molds for a stretchable tactile interface for display application [54].

## 3. Requirements of Rapid Prototyping

### 3.1. Printable Ink Preparation

With the advent of additive manufacturing in the 21^st^ century, a demand exists to establish novel inks or materials that are printable and functional. As most of the flexible/stretchable tactile sensor technology has adopted the drop-on-demand printing mechanism, it is necessary to fabricate the materials accordingly. This method relies on localized and controlled dispensing of materials on the substrate. The main challenge to tackle here is the ideal parameters that are required of the FSE ink [55]. It includes physical properties that could be controlled by density, particle size, viscosity, colloidal dispersion, and surface tension of the ink [56]. The optimum range of these parameters and how each property affects the print quality have been previously reported [57]. New materials comprising of metals, polymers, and composites are tuned to facilitate the FSE printing processes.

**Figure 6:**
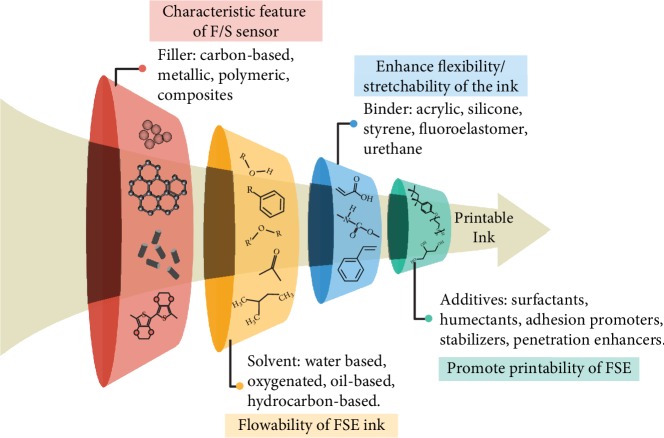
Typical components required for the formulation of a printable ink and the significance of their role [221].

In general, a typical FSE printable ink consists of 4 main components, filler, solvent, binder, and additive, as described in Figure 6. The filler determines the characteristic feature of the ink depending on the application. It could be made of metallic [58, 59], polymeric [60], and carbon-based material [61–64] or a combination of these. The flowability of the ink is determined by the solvent in which it is dispersed. Solvents dilute the other ink components, and water is a commonly used solvent [65]. It is important to examine the purity of the solvent so as to limit contaminants. Hence, it is responsible for the viscosity, surface tension, and drying rate of the ink. The viscosity of the ink can be adjusted by the addition of a polymeric thickening agent such as PVA [66]. In cases of multimaterial printing, it is important to choose different solvents and binder combination for the various layers as it can dissolve and damage the other layers in contact. The other important property all flexible/stretchable inks shall possess is the potency to maintain structural integrity on continuous flexing or stretching cycles. In the cases of stretchable inks especially, where higher amounts of strains are induced on the sensor device, the mechanical mismatch can cause cracks and delamination. This forces the conductive filler particles to distance, causing a higher resistance of the film. Increasing the concentration of the conductive filler material will lead to increased stiffness and reduced stretchability [67].

The third component of the ink addresses this issue by helping in binding the printed trace on the substrate material and supports in flexibility and stretchability of the sensor device [68, 69]. This binder further aids in homogenous dispersion of the filler material into the ink [70]. For instance, carboxylation of the CNTs favors effective dispersion in the solvent, which increases its potential to be used as a printable ink [71]. Binders help in cross-linking by mild drying or solidification by solvent evaporation. In some cases, annealing or exposure to radiation may be required for curing. To further characterize the ink, additives such as surfactants [72], adhesion promoters [73], and stabilizers are added according to the required rheological, stretchability, and wetting properties. The surfactant's role is to lower the surface tension of the resultant ink [74]. In certain cases, these surfactants uniformly distribute the ink nanoparticles by forming a protective layer on them and reducing the possibility of delamination of the printed trace [75]. The following chapters further explain the various components of the printable ink.

**Figure 7:**
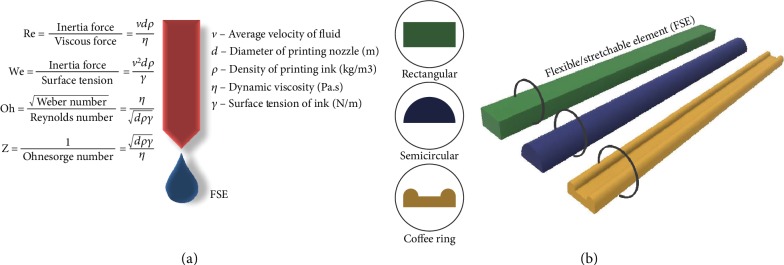
Important printability properties required for appropriate FSE printed trace. (a) Performance parameters of the droplet formation process. Reynolds number (Re), Weber number (We), and Ohnesorge number (Oh) together help to characterize *Z*, which helps determine the suitability of fluid for printing 1 < *Z* < 10, considered to be the optimal range of stable droplet formation [57]. (b) Various cross-section profiles of the printed trace depending on the viscosity of the inks.

One of the main criteria for a 3D printing method is the droplet formation [33]. It is a complex process governed by a few dimensionless numbers. Reynolds number (Re), Weber number (We), and Ohnesorge number (Oh) help characterize this process. The inverse of the Ohnesorge number is *Z* number, which assists in determining the stability of the droplet. The formulas are described in Figure 7(a). For lower *Z* values, droplet ejection is prevented due to viscous dissipation of the ink. For *Z* > 10, formation of a secondary (satellite) droplet following the primary drop is evitable [57]. Hence, after considering the various studies conducted over the years, 1 < *Z* < 10 is said to be the optimal printable range of *Z* for a stable drop formation [34, 57]. It is advisable for the size of particle exiting the nozzle to be 1/50 of its diameter, in order to prevent nozzle clogging [76]. The shear rate experienced by the ink through the nozzle can be estimated by

(1) $$\begin{array}{c} {\dot{\gamma }}_{W}=\left(\frac{3+b}{4}\right)\frac{4Q}{\pi R^{3}}, \end{array}$$

where *b* is the inverse of the shear-thinning power-law relationship, *Q* is the volumetric flow rate, and *R* is the radius of the printing head [77]. A minimum droplet ejection velocity is further needed to overcome the surface tension barrier at the fluid-air interface of the nozzle, which is expressed through the equation as

(2) $$\begin{array}{c} v_{\min}=\sqrt{\frac{4\gamma }{\rho d}}. \end{array}$$

The required minimum ejection velocity in terms of the Weber number is maintained >4 for a minimal printing value [34]. A minimum standoff distance (*h*_0_) of the nozzle printhead from the substrate is necessary to produce a stable printed trace. It is desired for the normalized standoff distance given as a ratio of standoff distance to the inner diameter (*D*_*i*_) to lie in the region 0 < *h*_0_/*D*_*i*_ < 0.21 [78]. The printed ink morphology is determined by the spreading and drying of the ink, influenced by the wetting properties as expressed by Young's equation in Section 4.1 [2]. Poor wetting leads to discontinuous feature formation as the ink would be incapable of maintaining contact with the surface. It is desirable to have a distinctive cross-section shape of the printed material. For the active element and electrodes especially, it is ideal for the conductive trace to have a rectangular cross-sectional profile, as shown in Figure 7(b), to possess certain electronic properties [79, 80].

### 3.2. Printer Requirements

The choice of a suitable 3D printing method before venturing into the research project or manufacturing is crucial. The compatibility between the ink, the substrate, and the printer plays a major role. The effect of concentration of filler material, the composition of the solvent, and hydrophobicity of the substrate surface have been discussed in the previous sections. However, to produce a well-defined and uniform printed feature, several other parameters need to be considered and optimized carefully. The feed rate and density of inks should be maintained at a constant. Printing accuracy and resolution of the printing are important as most of the sensor designs might require the features to be restricted to a particular dimension. In such cases, multilayer printing accuracy plays an important role. The high speeds of the printer should be able to maintain high-quality printing for several hundred cycles. This will ensure large-scale production at a reasonable cost.

**Figure 8:**
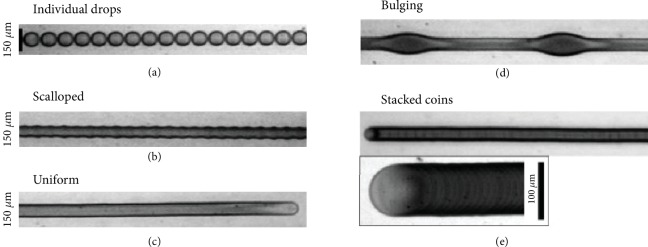
Different morphologies of the printed pattern with varied droplet spacing [66].

The final shape of the FSE printed pattern is affected by the contact angle of the drop on the substrate. This is influenced by the printing speed and droplet ejection. In typical cases, we require the droplets to overlap with adjacent drops while printing, to form a continuous feature [81]. Unstable drops occur if the droplet spacing is too small and the traverse velocities are low. The effect of varying drop spacing on the printed profile is shown in Figure 8 [66]. When the droplet spacing is greater than twice the drop's radius, isolated droplets are formed with a discontinuous printed feature. Likewise, the increase in the time delay between each droplet also varies the printing profile. As we decrease the droplet spacing, individual droplets start forming. After which, the droplets merge while retaining their individual rounded contact lines resulting in a scalloped line. Further decrease in the spacing will produce the uniform ideal printed pattern with the narrowest lines.

The final step in the printing process is the solidification of the deposited material on the substrate. In most cases, to accommodate to the needs of the printer, dilute solutions with a low concentration of particle suspensions are adopted. Hence, the solidification would be due to solute evaporation, where the quality of printing is influenced by the temperature of the substrate or the surrounding temperature it is exposed to. The solvent evaporates slowly at low temperatures leaving few active materials behind. At high temperatures, the solvent evaporates too quickly leaving aggregates of active material resulting in stacked coin formation as shown in Figure 8. This is when the time taken for single drop evaporation is less than the drop jetting period. Hence, it is important to experiment and find a suitable temperature for each printing material and modify the jetting frequency accordingly. The size and diameter of the nozzle (*μ*m) affect the morphology of the printed feature. A smaller nozzle diameter produces finer features with sharp edges. Likewise, a higher moving speed of the nozzle causes lesser particle deposition. However, if the printing speed is too low, particles get aggregates at the nozzle end. It is necessary to consider this trade-off and choose the optimum printing speed [82].

### 3.3. Flexible Device Mechanisms

**Figure 9:**
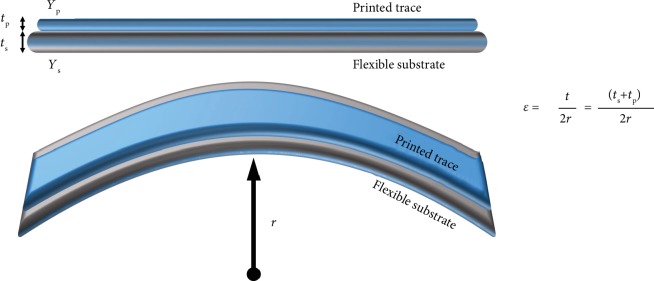
Mechanics of a flexible substrate and strain induced on its surface.

A common strategy to follow to make the sensor flexible is to make its printed layers sufficiently thin that it is able to be flexible and bendable [83, 84]. The strain induced on the surface of the bent sensor can be explained by the equation as illustrated in Figure 9, which is affected by the thickness (*t*) and the radius of curvature (*r*) [85]. Here, *t*_s_ and *t*_p_ refer to the thicknesses of the substrate and the printed trace on it. By reducing the thickness (*t*) of the sensor, the strain (*ε*) induced is lowered. Now considering materials with different elastic moduli (*Y*), where in many cases it is preferable for the substrate (*Y*_s_) to have lower modulus than the active layer (*Y*_p_), the strain on the top surface is reduced significantly following this equation:

(3) $$\begin{array}{c} \varepsilon =\left(\frac{t_{\text{s}}+t_{\text{p}}}{2r}\right)\frac{1+2\eta +\chi \eta^{2}}{\left(1+\eta \right)\left(1+\chi \eta \right)}, \end{array}$$

where *η* = *t*_p_/*t*_s_ and *χ* = *Y*_p_/*Y*_s_ [86]. Thus, having substrate and encapsulation layers with a low elastic modulus and reduced thickness will promote the bendability and flexibility of the sensor devices [87]. In the printing methods, the layer thickness can be conveniently controlled by adjusting the printing speed and nozzle diameter and by stable droplet formation.

Typically, a flexible element printable ink shall be able to maintain structural stability upon flexing. From the thermodynamic standpoint, the amount of work done *W* needed to delaminate the two surfaces depends on their specific surface energies and the interfacial energy between them [87]. This interfacial energy region between them is formed during the process of printing. An abrupt interface is formed when the film interaction is low, and it gives rise to high-stress gradients causing easier interfacial fractures. Hence, the binder component of the ink should be able to distribute the strain generated upon flexion without affecting the filler component [88, 89]. Researchers introduced nonconductive binders to impart flexibility in the inks, although they affected the conductive performance of the ink. To combat these issues, researchers started adopting conductive binders which enhanced the conductivity of the printable ink. Similarly, the adhesion promoters enhance the attachment between the printed ink and the substrate and mitigate the shear stress developed due to mechanical deformation [60, 90]. These promoters further enable chemical interaction and create a compound interface in which the adhesion is better for thin-layered films and poorer for thicker layers. This flexibility can be exploited to attain stretchability in the sensor devices which is discussed in the next section.

### 3.4. Stretchable Device Mechanisms

In general, the stretchable device mechanisms happen in two complementary ways where one is focussed on attaining new structural designs for high-performance conventional materials and the other relies on a new material approach or the usage of intrinsically stretchable materials for conventional layouts [83, 91]. These two forms could be implemented separately or as a combination of both. Stretchability not only is limited to the elasticity of the material but also relies on the adhesive strength between the various printed layers, the durability of the interconnects, and the thickness of every printed layer. The strategies discussed below equip the printed sensor with the counterbalance restoring force on applying tensile strain.

For one-dimensional (1D) stretchability, the wavy/buckling/wrinkle layout is preferred. To introduce this configuration, two types of mechanisms prevail: one is with prestretch and the other without prestretch. The first mechanism follows the prestretch-print-release strategy, where FSE is to be printed on a uniaxially prestretched elastomeric substrate. When released, the active element buckles and forms a wavy/wrinkled configuration orthogonal to the compressed direction [92, 93]. For the second mechanism, the wavy configuration is directly printed on the substrate. However, a print-stretch-release strategy was recently followed by using the helix electrohydrodynamic printer to fabricate secondary self-similar patterns of piezoelectric PVDF nano-/microfibers [94]. These formed structures bend and unbend upon stretching. For small strains, the relationship between the wavelength of the buckled configuration (*λ*), Young's modulus of the substrate (*Y*_s_) and printed layer (*Y*_p_), and the thickness of the printed layer (*t*_*p*_) is described by the equation as

(4) $$\begin{array}{c} Y_{\text{p}}=3Y_{\text{s}}\left(\frac{1-\nu_{\text{p}}^{2}}{1-\nu_{\text{s}}^{2}}\right){\left(\frac{\lambda }{2\pi t_{\text{p}}}\right)}^{3}, \end{array}$$

where *ν*_s_ and *ν*_p_ refer to the Poisson ratio of the substrate and the printed layer [95]. Notably, the wavelength of the buckled configuration is independent of the prestrain; however, to avoid delamination of the printed trace, large prestrains are to be avoided [96]. Similar to the uniaxial prestrain, biaxial prestrains induce herringbone geometry [97]. Another method is to induce cracks in the printed material by applying intentionally calculated strain and releasing them. This would align the cracks orthogonal to the stretching direction. It is important to note the design modifications that occur before and after the straining [98]. An alternative is to structure the substrate in a wavy/buckled configuration prior to the printing of the active layer. Silver nanoparticle ink was inkjet printed onto a wavy PDMS substrate which was created by a mold, to develop a stretchable conductor [99]. Similarly, 3D printing of the wavy-patterned substrate which was designed accordingly to accommodate the stretching and releasing cyclic deformation has been reported [100].

As we can understand that the wavy design configuration can provide stretchability up to 100% in one dimension, it is limited to ~10% when it comes to multiple directions [97, 101]. Hence, for omnidimensional device configuration, convoluted patterned geometry is preferred. In order to reduce delamination and increase stretchability, we have to localize the strains in the sensors by introducing optimized design patterns. Several inplane structures like horseshoe geometries have been fabricated and transferred to another stretchable substrate [102]. To avoid all the transferring and to reduce the fabrication steps, they could be directly printed using the widely available rapid prototyping methods. Nanoscale precise omnidirectional printing of silver microelectrodes using the direct ink writing showcased extreme stretchability and flexibility. Three-dimensional structures were obtained by printing in a layerwise sequence and could withstand up to 200 straining cycles without breaking [103].

In the case of stiffer functional materials with a higher elastic modulus, they are to be print patterned on an elastomeric substrate with lower elastic modulus. Design configurations such as mesh/serpentine/horseshoe/coiled/helical-spring/zig-zag are adopted to increase the stretchability of the printed sensors. Multiple iterations of the above-mentioned patterns further promote the stretching ability [104]. A graphene oxide aerogel-based sensor which was printed through microextrusion has been reported to yield more prominent mechanical properties with its serpentine patterns, compared to a typical straight-line patterning. The patterning of this aerogel sensor has led to a change in its compressibility and stagger resistance [105]. Similarly, fractal designs to yield space-filling geometries are adopted to accommodate the strains along various dimensions [106]. A comparison in the stretchability of the straight-line printing and sinewave track printing was carried out using conductive silver and PEDOT:PSS ink by printing on flexible and stretchable substrates. As expected, the printed sinewave patterns remained conductive for the lower radius of curvature than the straight-line pattern as it was more prone to cracks upon stretching [107].

The widely adopted island-bridge configuration is possible through the 3D printing technology as the printed circuitries connect the rigid island consisting of the required microelectronics to the stretchable interconnects. An ultrathin stretchable e-skin was developed by printing the connecting circuit on a tattoo transfer paper which was then transferred to various substrates depending on the application [108]. To avoid the transfer of printed components, a hybrid 3D printing technology which combined the direct ink writing with vacuum nozzle pick and place was presented. Functional and stretchable devices were developed using this method [109]. Typically, these configurations possess general stretchability but not local stretchability. Applying local strain at a point in the structure might lead to an unequivocal break. Hence, a number of such hierarchical structures are needed to be printed. Further structural patterns from 2D to 3D configurations that enhance the stretchability of the sensor design are reviewed in a recent publication, taking into account the theoretical and experimental research [110].

**Figure 10:**
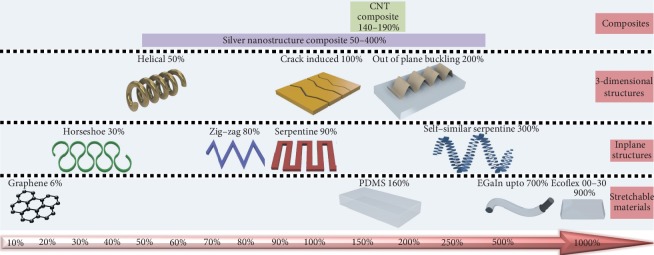
Various strategies to make the sensor stretchable placed according to their maximum elongations. Composites: CNT based [98] and silver nanostructures based [115]. 3-dimensional structures: helical [222], crack induced [223], and out of plane buckling [224]. Inplane structures: horseshoe [225], zig-zag [226], serpentine [227], and self-similar serpentine [94]. Stretchable materials: graphene [228], PDMS [229], EGaIn [230], and Ecoflex 00-30 [229].

The above-mentioned strategy involves a multistep printing procedure to achieve stretchability in the sensor devices, whereas a single step printing fabrication is possible with the next approach discussed below [111]. Moreover, it is well studied that elastomer-supported devices rupture or interconnects fracture at larger strains beyond a specific range [112]. Hence, the second strategy achieves stretchability by using elastic conductive material composites that are intrinsically stretchable where all of its components deform simultaneously and possess the conductive ability [113]. Some of these composites adopt the high-performance component of the functional material, which does not possess any intrinsic stretchability and embed them into an elastomeric matrix [114, 115]. The substrate material interactions explained in the previous sections apply here. The printable stretchable composites can be designed in different methods such as implanting, filling, infiltrating, blending, and synthesizing the fillers into the elastomers [116]. Stretchability can be imparted in the conductive materials by electrically anchoring the conductive fillers. Here, a printable elastic ink with a conductivity of 8331 S/cm is prepared with various silver conductive fillers of different sizes and structures and eutectic gallium-indium (EGaIn) particles as their electrical anchors. A stretching ability to withstand up to 700% strain was achieved by increasing the bonding strength between the filler particles and host polymer [117]. While one-dimensional or two-dimensional geometry-based configurations can generate a response to mechanical deformation, higher dimensional geometries are required to respond to multidimensional sensing.  Figure 10 sorts the design strategies to impart stretchability into the sensors and their maximum elongations as per the published literature.

## 4. Printing of FSE of Sensor and Material Requirements

Additive manufacturing technology presents a new era of tactile sensors due to its cost-effectiveness, scalability, and customizability. It is further essential for shape-conforming sensors to be flexible/stretchable without incurring any physical damage. Most of the conventional inorganic materials used in fabricating sensors are not adequate to satisfy the mechanical compliance due to their rigid features. For them to suit the 3D printing methods especially, new approaches in material designs and development of new functional materials are needed. Generally, a tactile sensor comprises of two distinct facets, the substrate base layer and the active functional layer. The substrate acts as the base and encapsulation layer and the active element, which is responsible for the transduced signal. Hence, the following section is dedicated to the commonly used materials in the fabrication of 3D printed flexible/stretchable tactile sensors.

### 4.1. Substrate

The substrate plays an essential role in any sensor technology as it determines the printability, flexibility, stretchability, and long-term durability of the sensor [118]. Owing to the nature of the flexible/stretchable substrates and other materials used to fabricate the soft, flexible/stretchable sensors, there are limiting factors for the processing conditions, especially for the printing methods. The determination of device fabrication methods depends on certain material properties such as allowable temperature, thermal stability, surface quality such as surface roughness and cleanliness, adhesion, radius of curvature, thickness, and transparency in some cases [83]. Processing temperatures are to be taken into consideration as the flexible/stretchable substrates have a much lower glass transition temperature than that of their rigid counterparts. The coefficient of thermal expansion (CTE) and the glass transition (Tg) temperature are important factors to consider when selecting the polymer material to evaluate their thermal stability [119]. At those mentioned temperatures, the chains of polymers relieve the energy stored during the operation [118].

**Figure 11:**
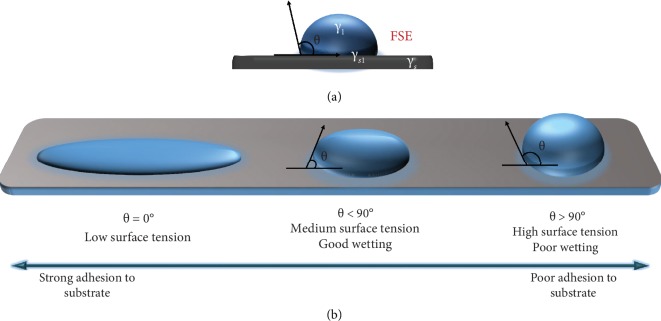
The wetting behavior of the ink droplet on the substrate. (a) *θ* is the contact angle between the printed drop and the substrate. (b) For higher *θ* values, the surface tension on the droplet dominates the engaging forces on the surface and it is more difficult to bond to the substrate.

Flexible/stretchable substrates tend to possess a higher coefficient of thermal expansion (CTE) than most inorganic materials. The resolution of the printed patterns is highly affected by the variation in the processing temperatures by causing overlay and alignment issues. Surface roughness is an essential factor to be considered, as the polymer substrates have uneven surfaces in general. Applying processes on them would increase their surface roughness, which is favorable in case of deposition of active material layers. Another factor affecting this interaction is the adhesion forces between the substrate layer and the active deposits which show their ability to remain bonded. Surfaces of some substrates are hydrophobic in nature, which repels the adhesion forces of the depositing printable inks. Hence, the surface energies of these materials play a crucial role. This property governed by Young's equation is given as *γ*_*s*_ = *γ*_*s*1_ + *γ*_1_cos*θ*, where *γ*_*s*1_ is the interfacial free energy, *θ* is the angle between the contacted surfaces, *γ*_*s*_ and *γ*_1_ are the surface energy of the substrate and the ink droplet, respectively. The condition to maintain good adhesion between both is when *γ*_*s*_≫*γ*_1_, as depicted in Figure 11. *θ* < 90° indicates good wetting, and *θ* > 90° suggests poor wetting [56]. The addition of adhesion promoters which had been previously discussed in the ink formation section also improves the adhesion by changing the chemistry of the ink, which reacts with the surface to form strong interfacial bonds.

There are two widely used approaches in improving the adhesion of the inks, especially metal nanoparticle ink. One modifies the ink by stabilizing it with surfactants or additives, which uniformly distributes the nanoparticles by forming a protective layer on them. But this hinders the full potential conductivity of the deposited structures. The other is to condition the surface of the substrate by increasing the surface energy. Physical-chemical modifications can be made by exposing the substrates to ultraviolet, ozone [120], plasma treatments [121], flame treatment, and other similar techniques. Chemical modification attempts have been made using self-assembled layers [122]. The surface tension of the substrates, which affect the wetting conditions, can be modified by electrical means with the applied electrical field. This applies to conditions only when there could be a potential difference between the substrate and the deposited active material droplet, separated by an insulator [123]. Recently, a direct printing method assisted by electrowetting employed voltages between the nozzle and the substrate material. By varying the electric field strength, they were able to print traces of width from 50 to 720 *μ*m [124]. This is important in the printing process as it increases the ink adhesion by improving the interfacial interaction between the substrate layer and the active layer.

Therefore, the typical requirements of the substrate material to fabricate the flexible/stretchable tactile sensors by the printing methods include low processing temperatures, high surface roughness, and low coefficients of thermal expansion (CTE). In the case of stretchable sensors, the main requirement is to make them elastically and electrically functional while pushing them to their stretching limits. This curbs the choice of possible materials. The widely used organics exhibit low electronic performance to the extensively used metals and other semiconductors, restricting their scope of application.

The commonly used FSE substrate materials are categorized into silicone elastomers, polymers, and others. Among the silicon-based organic polymers, polydimethylsiloxane (PDMS) has received an overwhelming response due to its promising features such as intrinsic stretchability (low elastic modulus of 1.84 MPa), transparent appearance, nontoxic nature, high thermal stability, chemical resistance, hydrophobicity, commercial availability, and easy processability. Due to the siloxane (Si-O) linkages of the cross-linked molecular chain of PDMS, the divalent oxygen atoms help in chain extension between the Si atoms. This makes it recoverable after the mechanical deformation and allows it to exhibit stretchability up to 1000% and elastic regimes up to *γ* ≈ 700% strain. In addition to these properties, the variable viscosity of the polymer along with its cross-linking ability makes it easily patternable into pyramids or grooves or into any other modifications in the surface microstructures leading to better sensor performances [125].

Ecoflex™ rubber is an off-the-shelf silicone that is widely used in these applications. It is highly stretchable and available at different levels of viscosity and tensile strength. It is a skin-safe biodegradable silicone [126] with a low elastic modulus of 0.07 MPa. Dragon Skin is one such stretchable silicone elastomer. Polyimide (PI) exhibits high thermal, chemical, and creep resistance. Due to this property, it has gained popularity as a flexible substrate in the field of flexible electronics. However, its intrinsic orange color has inhibited its scope in transparent devices. Nevertheless, attempts had been made in developing transparent versions of the orange-tinted polymer. An additive manufactured thin and flexible microsensor was developed to measure shear stress and pressure, utilizing polyimide as a substrate layer [127]. Polyethylene terephthalate (PET) is often used as a substrate film for flexible tactile sensors. Due to its high modulus, as shown in Table 4, it cannot be stretched but has other benefits such as transparency up to 90%, high creep resistance, and suitable surface energy that allows printability of conductive inks. An inkjet-printed soft tactile sensor on PET substrate material was coated with hydrogel and mounted on surgical tools [128]. Other conventional polymer substrates would include polyesters such as Polyethylene Naphthalate (PEN) which possess hydrophilicity, thermoplastic polylactic acid (PLA) [129], polyurethane (PU) [130], and poly(styrene-b-butadiene-b-styrene) (SBS) [131].

Another material such as silk which is biocompatible, biodegradable, and can endure irregular deformation has been used in bioelectronics [132]. Silk-based inks have been formulated for other applications, but they are yet to enter the spectrum of fabricating flexible tactile sensors through additive manufacturing technologies [133, 134]. Similarly, the paper is an inexpensive, porous, flexible, lightweight, and renewable resource that has shown the potential to be a suitable substrate for flexible tactile sensors [135–137]. Paper was used as one of the electrodes in the fabrication of an inkjet-printed bimodal strain and a pressure sensor. This paper was soaked in glycerinum to enhance its substrate properties so that the printed ink layer would adhere to it. It was further tested under 900 bending cycles to confirm its mechanical stability and conductive degradation [35]. Other types of flexible substrates include metallic foils and thin inorganic glasses. Metal foils sustain high temperatures, possess good chemical resistance, and cater to the deposition of inorganic materials [138]. However, the surface roughness and high costs associated with it curb its usage in flexible sensor fabrication.

### 4.2. Active Elements

The active FSEs in some cases are functional inks which include CNTs/graphene solution, nanoparticle/wire/flake inks of conductive metals, several conductive polymer-based inks, dielectric materials, and other composites. The important constraints with these materials are its particle size, solubility, colloidal dispersion, viscosity, flowability, surface tension, and density of the solution [56]. There are studies on the printable elastic inks with various conductive fillers in different polymer matrices. Important parameters required for selecting the appropriate active element of the printable ink are listed in Table 5.

#### 4.2.1. Metallic Ink

Silver-based inks with fillers such as silver flakes, nanoparticles, and nanowires prove to be the desired choice for printed electronics as it provides attractive and necessary features such as resistance to oxidation and high electrical conductivity [58, 139]. Silver's conductivity dependency on the different printed substrates was studied and characterized in a recent study [140]. Highly stretchable and printable inks are being developed using silver nanoparticles [103, 141]. Copper-based inks limit its application due to the oxidation that takes place after printing. To overcome this problem [142, 143], a research group has used hydrazine treatment and reported a well-sintered microstructure with low resistivity [144]. A major disadvantage with the metal nanoparticle inks is its precipitation and agglomeration of particles, which causes nozzle clogging. To avoid this, the metal colloids can be stabilized by adding dispersants along with its formulation. Another problem is the adhesion of NP inks with the polymer layers; it is relatively weak as compared to the carbon-based materials.

The other commonly used type of metallic ink is a liquid metal such as the eutectic gallium-indium (EGaIn) liquid metal alloy which maintains its liquid state at room temperature [117, 145]. It does not possess any elasticity, but it is compatible with stretchable systems. To make this liquid metal printable, various rheological modifications have been implemented such as promoting oxide build-up through continuous stirring for a long period of time. This increases the surface wettability of the liquid metal which enhances the adherence on the substrate [146]. It has gained popularity in stretchable electronics due to its strong adhesion on substrates and its excellent electrical conductivity [78]. Printing of EGaIn further leads to oxidation of the metal alloy and accumulation of it at the nozzle tips. To combat this problem, an oxygen-free environment was chosen to print liquid GaIn alloy, for which the pendant drop method was used to characterize the alloy extruded in a nitrogen-filled glovebox. Electrowetting assisting selective printing of EGaIn alloy by the direct-write printing method was recently reported [124].

#### 4.2.2. Carbon-Based Ink

Owing to its superior electrical and mechanical properties, carbon-based materials like graphene [64, 147, 148] and CNT [61, 149] have been prominently used as an FSE. CNT films exhibit piezoresistive property. A single CNT shows high sensitivity to strain with a gauge factor > 1000, but it gets more complicated to construct it on a large scale. It is resilient to deformation showing high tensile strength in the order of a hundred GPa [150] and has high mobilities enabling them to operate at the low operating voltage [151]. Having an outstanding electroconductibility, inplane stretchability up to 25%, high modulus, thermal conductivity, and optical transmittance > 97%, graphene has secured its place in this field [152]. Printable graphene inks are prepared through the mixing of an elastomeric solution with graphene powder dispersed in a solvent, which could produce features as small as 100 *μ*m and up to a few hundred layers [80, 153]. Electrical conductivity of inkjet-printed reduced graphene oxide (RGO) on a polyimide substrate can be significantly improved by annealing it through the laser radiation technique [154]. Apparently, the giant graphene oxide (GGO) sheets show typical shear-thinning behavior which makes it suitable for extrusion-based printing techniques. GGO direct printed through a 200 *μ*m nozzle exhibited self-assembly behavior into close-packed lines through the *π*‐*π* conjugation effect and hydrogen bonding [19].

#### 4.2.3. Polymer Ink

Having mechanical similarity with the FSE substrate polymers, the polymer-based inks have been widely studied. Poly(3,4-ethylenedioxythiophene)-based polymers exhibit high thermal stability, good optical transmittance, solubility in various solvents, and tunable conductivity. The commonly used poly(3,4-ethylenedioxythiophene) polystyrene sulfonate (PEDOT:PSS) is a universally available commercialized polymer [60]. However, due to its intrinsic hardness, once dried, the film becomes brittle. To overcome this, small molecules such as xylitol or sorbitol or capstone [60, 120] could be added to increase the plasticity of the polymer or could use a porous substrate to promote adhesion.

A widely used FSE material for piezoelectric sensors is polyvinylidene fluoride (PVDF) and its copolymer, polyvinylidene fluoride-trifluoroethylene (PVDF-TrFE), due to its high dimensional stability and piezoelectric coefficient. The disadvantage is with its indistinguishable pyroelectric effect, which calls for protection from thermal interference. Ionic liquid (IL) has recently gained popularity due to its low viscosity, low volatility, good chemical, thermal stability, high contrast wettability control, solubility in various organic solvents, and high ionic conductivity [72]. 1-Ethyl-3-methylimidazolium tricyanomethanide is an imidazolium-based ionic liquid that exhibits a conductivity of 18 mS cm^−1^ and a low viscosity of 18 Pa s [155]. Polypyrrole (PPy) is a pseudocapacitive, biocompatible conducting polymer having chemical stability with alkalines. However, due to its insolubility in solvents, its applications and processing techniques have been limited. Hence, colloidal solutions of PPy nanotubes/nanorods are prepared as a promising FSE [156]. Its potential as a supercapacitor electrode has been published as PPy nanowire-based material exhibited large specific capacitance and cyclic stability [157, 158].

Ionically, conductive and printable hydrogels are biocompatible and stretchable [159, 160]. They conduct through ions, and they show a change in resistance or capacitance upon the application of mechanical stimuli [161, 162] Due to the presence of reversible hydrogen bonding, they exhibit self-healing properties [163]. A self-healing, stretchable, and printable hydrogel revealed that the printing parameter such as applied pressure, nozzle diameter, temperature, and moving speed was controlled accordingly to produce the appropriate printing hydrogel trace [41, 164]. This hydrogel was printed on a gelatin membrane, used as a sacrificial layer, as it was found that the surface properties of common substrates like PDMS/glass could not accommodate it. A 3D printed, multifunctional thermal, and pressure-responsive double-network hydrogel shows potential application as an artificially intelligent skin. The heating of the hydrogel above its volume phase transition temperature (around 30°C) lowers its viscosity exhibiting a shear-thinning characteristic that aids its printability [165]. In addition, the conductive layers were microstructured to make it responsive to small stimuli. Other potential polymers are Polyaniline (PANI) [166], polyphenylene vinylene (PPV) [167], thermoplastic polyurethanes (TPU) [46], etc.

#### 4.2.4. Composites

The poor processability issues of composite materials encountered with the traditional techniques could be circumvented by implementing the additive manufacturing processes [129]. The electronic properties of the printable composite (*σ*—electrical conductivity) materials are largely dependent on the volume fraction of the doped conductive filler materials, *σ* = *σ*_*o*_(*V*_*f*_ − *V*_*c*_)^*s*^, where *σ*_*o*_ refers to the scaling factor, *V*_*f*_ is the volume fraction of the conductive filler, *V*_*c*_ is the percolation threshold, and *s* is the conductivity exponent [168]. The fillers can be chosen from carbon-based materials, metallic filler materials, or other conductive organics [114]. The morphology, distribution, geometry, and adhesion of the filler particle in the composite matrix play a role in determining the performance of the printed conductive composite [169]. For effective and a homogeneous mixture, magnetic or ultrasonic stirring can be utilized. Improved stretchability and cyclic stability are attained through strong bonding between the two materials [170]. A highly stretchable and printable composite impregnated with silver flakes and MWCNTs as filler material exhibited high conductivity of 5710 S/cm at 0% strain [171].

It is recommended to maintain the concentration of the filler material around the percolation threshold (PH) to obtain a high gauge factor in strain sensors [172]. Lower concentrations than PH will lead to dimensional separation between the particles leading to an exponential increase in tunneling resistance. Higher concentrations than PH will decrease the tunneling effect and hence a significant decrease in the gauge factor [173, 174]. The ionic transport properties of a 3D printable polymer/ionic liquid composite were discussed in a study recently. It was found that the extent of cross-linking and polymerization of the composite greatly affected the sensitivity of the printed sensors [72]. In the mechanical aspect, large concentrations of filler material lead to augmented stiffness and bare lower strain at break. There is a rise in the 3D printable carbon-based composites due to their high conductivity, flexibility, and high anisotropic property [175]. When the CNT network is dispersed in an elastic substrate, it exhibits two types of resistance. One is its own intrinsic resistance, and the other is the dominating innertube resistance; both of these give rise to the strain sensing phenomenon [176]. In another case, dichloromethane was adopted as a dispersing medium for the preparation of intrinsically conductive PLA/CNT composite which was direct 3D printed via liquid deposition modeling [177]. The purpose of composite materials is to combine the desired properties to two materials and developing a matrix favorable to the application. Recently, 3D printable hydrogel-PEDOT:PSS composite was developed by freeze-drying the PEDOT:PSS solution and mixing it with PEGDA, a photocurable base [178].

Both organic and inorganic materials required to maintain high capacitance by acting as an insulating layer in low-voltage applications are explored [179]. While the organic ones have already been established in the printing processes, the inorganic ones are yet to make their way. There are plenty of commercially available dielectric inks with varied dielectric strengths and optical transmittance. Materials such as polydimethylsiloxane (PDMS) dielectric [125], polyvinyl alcohol (PVA), Polyaniline (PANI) [166], poly(4-vinylphenol), poly(methyl methacrylate) (PMMA), [180] barium titanate (BaTiO_3_) [181], polystyrene, terephthalate, and polyimide (PI) [182] are commonly used for additive manufacturing of dielectrics. Ionogels with intrinsic stretchability exhibit a high capacitance of ~10 *μ*F/cm^2^ and are used in several applications [183]. It is further studied that patterned dielectric surfaces result in better pressure sensitivity [184].

## 5. Printability of Flexible/Stretchable Tactile Sensors

Among the various essential functions and properties of our human skin, the sense of touch plays a major role. The ability of our skin to distinguish a minute pressure as low as 5 Pa and further withstand a high mechanical force poses challenges to engineering it. Attempts have been made to develop a fully functional electronic skin and an epidermal electronic system using additive manufacturing techniques. These systems have multiple substantial applications in the field of biomedical engineering and health monitoring [185]. Tactile sensors equip us with the information on the mechanical interaction of the sensor with the object through physical contact. Upon detecting and measuring this given property, the signal is transduced to an electrical output. To further understand the tactile sensors, its physical principles, sensor attributes, materials required for fabrication, design layouts, and promising applications are discussed in the chapters below.

### 5.1. Printable Pressure Sensors

Early developments of tactile pressure sensors focussed on the various transduction principles. For the printable flexible/stretchable tactile sensors, the mechanical stimulations like pressure, force, and vibration are converted through 4 main mechanisms such as piezoresistivity for strain gauges, the capacitance for structures sensitive to compression, piezoelectricity for voltage variations due to mechanical deformation, and triboelectricity for vibration-induced potential difference [186]. We also have the field-effect transistor- (FET-) based tactile sensors that use an electric field to control the flow of current. However, most of these high sensitivity devices are rigid in nature. To overcome this problem, organic FET utilizing stretchable polymers have been utilized [187], whereas this being fabricated through printing technology is still a challenge [188].

#### 5.1.1. Piezoresistive

**Figure 12:**
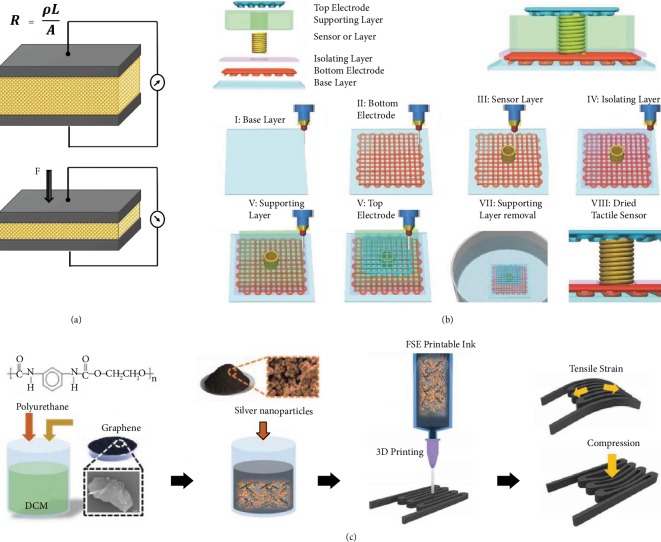
Printed flexible/stretchable piezoresistive pressure sensor. (a) Schematic illustration of the piezoresistive transduction principle. (b) Printed stretchable tactile sensor with the multimaterial fabrication: (A) schematic of different layers of a tactile sensor; (B) 8 sequential steps of the 3D printing process [231]. Reproduced with permission. Copyright John Wiley and Sons, 2017. (c) Direct printing of nanocomposite printable inks for piezoresistive sensor applications [232]. Reproduced with permission. Copyright John Wiley and Sons, 2018.

The principle of piezoresistive-based pressure sensors is based on the piezoresistive effect. Piezoresistive material transduces the mechanical variations into detectable resistive or conductive changes, measured by an external electrical circuit. It is the common transduction principle, due to its high sensitivity, uncomplicated structure, low cost, wide range of detection, low energy consumption, and manageable readout mechanism. The relative change of resistance is written as shown in Figure 12(a), where *R* is resistance, *ρ* is resistivity, *L* denotes the length, *A* refers to the cross-sectional area of the conductor, *ν* is Poisson's ratio, *ε* is applied strain, and *G* is gauge factor (GF). Hence, it can be inferred that the resistance is dependent on the geometry and resistivity of the sensing material.

This piezoresistivity is caused by the stress that modifies the band-gap which in turn alters the mobility of the charge carriers. This is implemented either by modifying the contract state of the conductive materials or through tunneling effect [149]. The former is observed in silicon, CNT, and graphene-based piezoresistive sensors. The latter is observed in conductive composite materials based on conductive nanoparticles, nanowires, nanotubes, and flakes as current tunneling through nanogaps [82]. The disadvantages associated with these types of tactile sensors are their dependence on temperature, stability issues, and hysteresis effect. A highly elastic strain sensor performing under the piezoresistive mechanism was 3D printed by the fused filament fabrication (FFF) technique. The thermoplastic polyurethane and multiwalled carbon nanotube composite were extruded into filaments to make it compatible with this printing technique. The sensor possessed high gauge factor of 176 under 100% strain [189].

#### 5.1.2. Piezoelectric

**Figure 13:**
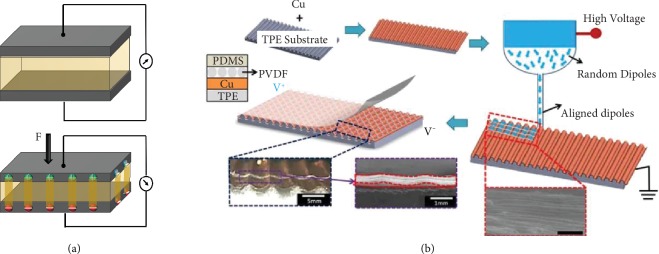
Printed flexible/stretchable piezoelectric pressure sensor. (a) Schematic illustration of the piezoelectric transduction principle. (b) Step by step fabrication of PVDF fibers deposited on the 3D printed wavy substrates to produce the self-powered pressure sensor [100]. Reproduced with permission. Copyright Springer Nature, 2017.

Piezoelectric sensors respond to mechanical stimuli by electric polarization and production of voltage. This transduction phenomenon is called piezoelectricity. Dipole moments are observed from the deformation of the oriented crystalline structures. Piezoelectric materials have a property of electromechanical coupling with the generation of electric charge under mechanical deformation. The energy conversion efficiency of piezoelectric materials is evaluated by the piezoelectric coefficient (*d*_33_), which is the ratio of open circuit charge density to the applied stress (*C*/*N*) units [190]. This type of sensors shows rapid response, high sensitivity, low power consumption, and self-powering/energy harvesting characteristics. Conventional piezoelectric materials such as inorganic ceramics, single crystals, and lead zirconate titanate (PZT) possess high *d*_33_ but are too brittle for the flexible/stretchable tactile sensors. Contrarily, piezoelectric polymers such as poly(vinylidene fluoride-trifluoroethylene) (PVDF-TrFE) have mechanical flexibility, chemical stability, uncomplicated processing, biocompatibility, and low cost. The drift of sensor output over time and its susceptibility to temperature are the major disadvantages in this type of sensors. Recently, PVDF piezoelectric films were printed and activated using an FFF printer integrated with corona poling process. These sensors were tested on a dynamic load range from 5 to 45 N for 50 cycles [50]. A self-powered pressure sensor was fabricated with piezoelectric fibers. The near-field electrospinning process was used to deposit the PVDF fibers on the 3D printed substrate as shown in Figure 13(b) [100].

#### 5.1.3. Piezocapacitive

**Figure 14:**
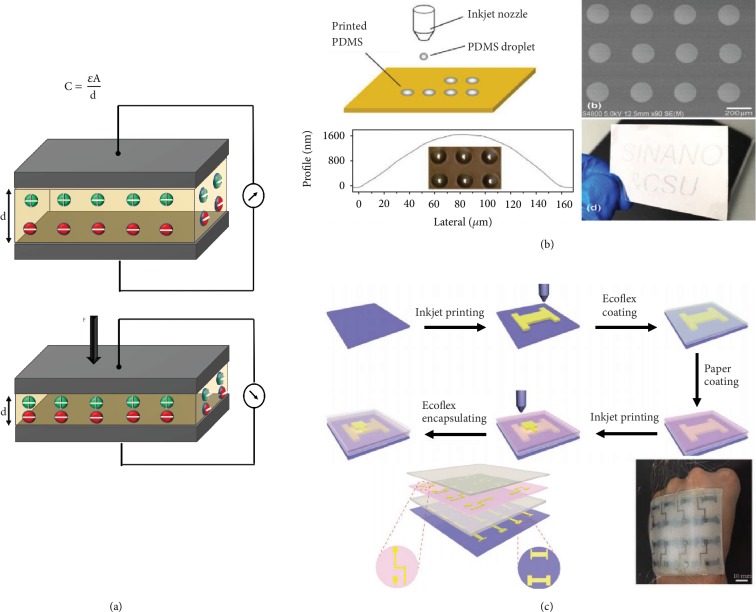
Printed flexible/stretchable piezocapacitive pressure sensor. (a) Schematic illustration of the piezocapacitive transduction principle. (b) Microstructured PDMS inkjet printed and sandwiched between ITO-coated glass [198]. Reproduced with permission. Copyright AIP Publishing, 2017. (c) Schematic of the fabrication process of a bimodal e-skin with 4 × 4 pixel sensor and its strain distribution mapping [35]. Reproduced with permission. Copyright John Wiley and Sons, 2019.

As for capacitive sensing, it relies on the charge storage and the capacitance change upon deformation. The capacitance, *C*, of a parallel plate capacitor is as shown in Figure 14(a), neglecting the fringe effect. We use *ε* to represent the permittivity of the dielectric material, *d* being the distance between the two plates and *A* the overlapped area of the two plates. The gauge factor of a capacitive strain is given by (Δ*C*/*C*0)/*ε* or (Δ*C*/*C*0)/*P*, and here, *ε* represents the strain and *P* the intensity of pressure. Mechanical deformation brings the electrodes closer and induces changes in *d* and *A*, resulting in changes in the capacitance of the dielectric medium. Apart from the traditional parallel plate configuration, an interdigital configuration is widely incorporated in capacitive sensors [191]. An aerosol jet printed interdigitated capacitive sensor, with a sensor thickness of 0.5 *μ*m, was fabricated for touch sensing applications [192]. The capacitance-based sensors are advantageous in various aspects such as high strain sensitivity, temperature independence, low power consumption, and low signal to noise ratio. Sensitivities of these types of sensors can be improved by addressing the compressibility of the materials by using low modulus materials. Likewise, altering the surface of the electrode or dielectric layer with patterns and microstructures like dome-shaped [193], pyramidal [194], or interconnected hollow-spheres [195] shows improved sensitivity [196, 197]. N-Butyl acetate diluted PDMS was fabricated as a microstructured dielectric layer by inkjet printing on an indium tin oxide- (ITO-) coated glass substrate as shown in Figure 14(b). It exhibited a high-pressure sensitivity of 10.4 kPa^−1^ and showed the feasibility to be printed on a flexible PET film [198]. High susceptibility to electromagnetic interference can modify the fringe fields of the capacitor [199]. Cross-talk between the taxels and complex circuity is the disadvantageous characteristics of capacitance-based sensors.

#### 5.1.4. Triboelectric

**Figure 15:**
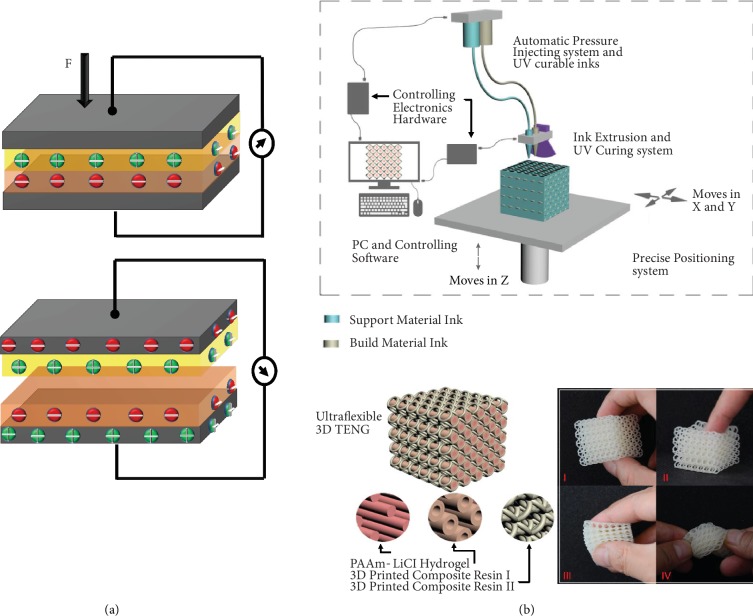
Printed flexible/stretchable triboelectric pressure sensor. (a) Schematic illustrations of the triboelectric transduction principles. (b) Fabrication procedure of the ultraflexible triboelectric nanogenerator of different sizes and intricate structure [202]. Reproduced with permission. Copyright Elsevier, 2018.

Triboelectric sensors function on the basis of the coupling effect of contact electrification and electrostatic induction [186]. While this mechanism is still under investigation, it is understood that when two different materials are rubbed with each other, electrical charges are induced on their surface. The polarities between the two materials affect the amount of electrical charge generated. Upon a mechanical deformation, when the two surfaces come in contact, opposite charges are induced on their surface. Once the deformation is released, the two surfaces automatically separate. Owing to the air gap between them, the opposite charges on surfaces create a potential difference. These are mechanical energy harvesting devices; in other words, it enables to monitor touch action without the necessity of external power supply [200]. Triboelectric generators demonstrate excellent compatibility with the printing fabrication techniques as its fundamental structural components are printable without compromising on the device performance. A fully printed triboelectric nanogenerator was fabricated and used to analyze the various vibration types. It explains the advantages and the possibility of printed triboelectric sensors [180]. A 3D tribo-nanoprinting technology provides a cost-effective way to produce 3D triboelectric architecture [201].  Figure 15(b) shows that an ultraflexible triboelectric nanogenerator was 3D printed. It demonstrated a decent output of 10.98 W/m^3^. It was embedded into a shoe sole and developed into a self-powered LED lighting shoe [202].

### 5.2. Printable Strain Sensors

**Figure 16:**
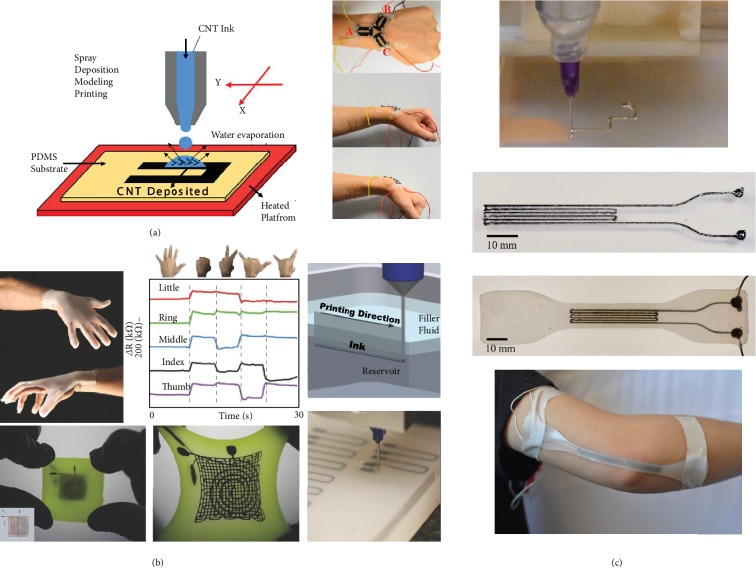
Printed flexible/stretchable strain sensors. (a) Printing fabrication steps of the piezoresistive strain sensor and its application on the wrist [205]. Reproduced with permission. Copyright American Chemical Society, 2018. (b) Embedded 3D printed strain sensor and its application as a glove [41]. Reproduced with permission. Copyright John Wiley and Sons, 2014. (c) Liquid metal alloy EGaIn printed and encapsulated to perform as a strain sensor to detect the elbow flexion and extension [207]. Reproduced with permission. Copyright IEEE, 2018.

Strain sensing is performed through two mechanisms: changes in resistance or capacitance occur by geometric alteration or by changes in internal conductivity. The conventional strain sensors produced by film transfer or solution casting are restricted to a basic geometrical shape such as a rectangle and a limited strain sensitivity in one direction [203]. Hence, studies on additively manufactured stretchable strain sensors are aimed at accommodating high levels of strains in multiple directions with arbitrary configurations and commendable sensing properties. A microrandom ridged-substrate-based strain sensor with a composite film thickness of 500 nm and stretchability up to 30% was fabricated through inkjet printing. In the graphene/ZnO composite, connectivity between graphene flakes was enhanced due to the addition of ZnO nanoparticles [204]. In the stretchable composite-based strain sensors, the change in resistance or capacitance is linear at small strains as it has the elastic percolations between the conductive particles. It further increases rapidly once the strains cross the critical point. For instance, a highly stretchable piezoresistive strain sensor was fabricated with controlled sensitivity using a SDM printer as illustrated in Figure 16(a). MWCNT ink was prepared for the functional layer, and acid-treated PDMS was used as the substrate. The CNT ink was printed in a layer by layer fashion following the CAD-designed geometry with micrometer thickness. The sensors exhibited stretchability up to 45%, a gauge factor of 35.75, and a drift of only 20% after 1000 loading cycles, which has been used to monitor various human motions [205].

Conductive carbon black particles suspended in silicone oil, known as carbon grease, are favored as a functional ink in the additive manufacturing technology due to its strong shear-thinning property. The apparent viscosity of this ink lowers with the increase in the shear rate as it undergoes the extrusion through the fine nozzles. Embedded 3D printing seized these advantages of the conductive carbon grease and developed a three-layer stretchable strain sensor. The geometries of the sensors were varied according to its application. For instance, by adjusting the nozzle size to 410 *μ*m, a pressure of 50 psi, and altering the printing speed from 0.5 mm/s to 4 mm/s, it can fabricate a sensing glove to map the movement of each finger at five different positions (Figure 16(b)). It was further found that increasing the printing speed decreased the cross-sectional area of the printed feature which leads to an increase in its resistance [41].

Another type of strain sensor exploits the liquid metals and their alloys, having melting points lower than the room temperature. These conductive liquid metals or their alloys are embedded within microfluidic channels, where the resistance changes according to the geometric deformation of the liquid channel. The oxidation problems associated with the printing of liquid metal alloys have already been explained in the previous section. EGaIn comb capacitor was produced by depositing the liquid alloy as individual droplets of 340 *μ*m diameter on Ecoflex substrate by microcontact printing technique forming a conductive microfluidic channel [206]. Recently, a stretchable and wearable resistive strain sensor was printed by direct extrusion and was encapsulated with silicon layers. A liquid metal paste of eutectic alloy ultrasonicated with nickel nanoparticles was specially prepared to cater to the need of 3D printing fabrication technique in this paper. This strain sensor was used to measure the joint flexion angle of the hand at the elbow as shown in Figure 16(c) [207]. The high surface tension and other anomalies associated with the liquid metals and their alloys make the fabrication process more complicated. Hence, other conductive solutions are sought after. Poly(ethylene glycol) glycerol gel electrolyte was used as the conductive layer in a stretchable multicore-shell strain sensor printed with custom-designed coaxially placed nozzles. A modified version of commercially available silicone, Dragon Skin, was used as a dielectric layer and the encapsulant. The sensing characteristics of this strain sensor could be tuned by varying its geometry, like fiber length, diameter of the fiber, and thickness of each layer. This was possible to achieve by modifying the nozzle designs and the printing parameters [39].

## 6. Applications

**Figure 17:**
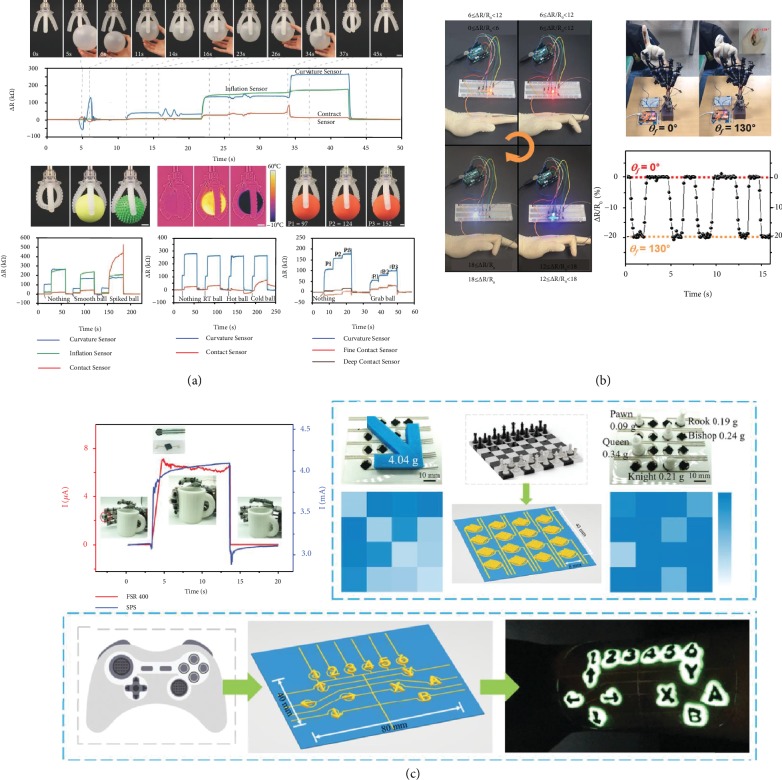
Highlights of the promising applications of printable flexible/stretchable tactile sensors. (a) Soft robotic grippers innervated with sensors grasp objects of various size, shape, surface, and temperature [40]. Reproduced with permission. Copyright John Wiley and Sons, 2018. (b) Detection of human motion under the applied strain and remote control of the robotic finger [149]. Reproduced with permission. Copyright American Chemical Society, 2018. (c) The stretchable piezoresistive array for various applications, such as transfer of cup using the control of the robotic arm, shape detection of an arrow-shaped object, and application in the chess game and as a stretchable gaming pad console [212]. Reproduced with permission. Copyright John Wiley and Sons, 2018.

Additive manufacturing of flexible/stretchable tactile sensors is creating new opportunities in wearable electronics [1], epidermal electronic systems [2], human-machine interfaces, soft robotics, education [208], and other biomedical devices (Figure 17). In wearable and epidermal electronics, these sensors are used for gait detection and analysis [209], expression recognition, swallowing and blinking motion monitoring, voice monitoring, diagnosing conditions like Parkinson's, sign language translation, body gesture [20], and posture identification. These sensors are used for real-time health monitoring, especially for patients with chronic diseases and who require continuous monitoring. For sensors with lower pressure sensing range < 10 kPa, high sensitivity is managed for tiny tactile perceptions of pulse sensing on the wrist. A wireless pressure and temperature sensor embedded into a shoe insole for continuous monitoring were printed using a multimaterial 3D printing platform. As the Internet of Things (IoT) is prevalent, it urges for fitness tracking during various activities to maintain a health record and sports performance. A carbon fiber-filled conductive silicon rubber composite was used to print a strain sensor and analyze the wrist motion [210]. Inkjet-printed strain sensors were attached to compression wear that is tightly attached to the body. These stretchable sensors provided the pressure and strain information on the legs [211]. Detection of human motion is crucial for personalized rehabilitative therapy in the case of patients with disabilities.

In human-machine interfaces and robotics, they are used to convey commands, electronic signature, haptic interfaces, object identification, soft robotics, and texture recognition and control robotic arms [149, 212]. A multitouch capacitive sensor was developed to remotely control and send various commands to the other interfaces. An integrated stretchable capacitive sensor and elastomeric actuator were fabricated using the direct ink writing technique. This actuator cum sensor was able to detect and compress force ~2 N. It was able to encode and read the haptic information. The pressing sequence on the sensor was recorded by the microcontroller and played back in the same order. Hence, tactile sensor input detection was responded with a kinesthetic haptic output [161]. An ear strap was fabricated to control the volume and switching between music tracks [213]. It helps in forming a closed loop by enabling the robot to understand the environment better with the sense of touch. Recently, a carbon aerogel-based 3D printed strain sensor was developed for logic identification of shape conversions [214]. In the biomedical field, it aids as a functional skin for prosthetics by added sensing capabilities [215]. In total knee replacement (TKR) procedures, attempts were made to embed smart sensors to monitor the load distribution and kinematic performances [216]. It is essential for the sensors to be customized according to the dimensions of the bearing so that they do not interfere with the critical geometries through additive manufacturing. The ease of fabrication of sensors that this technology offers has broadened its scope in the education systems as well. Laboratory modules have been conducted for students introducing them to the emerging trends of additive manufacturing [208].

## 7. Summary and Discussions

This review highlights the latest advances in the field of printed flexible/stretchable tactile sensors. The various tactile sensing mechanisms involved working principles such as the piezoresistive, capacitive, piezoelectric, and triboelectric, with emphasis on force, pressure, strain, and vibration in this paper. We focus on the essential features of tactile sensors such as high sensitivity, linearity, fast response, low hysteresis, durability, repeatability, and flexibility/stretchability. The fabrication strategies are to maintain the integrity of material properties and at the same time to meet the requirement of quality printing. Taking advantage of the technological trend, it is crucial to work towards solving the barriers in the way of fabricating these sensing devices. Compared to the nonadditive fabrication techniques, such as photolithography, 3D printing is more cost-effective as it does not require expensive facilities and mask fabrication. Moreover, since complex processes like etching are not involved, there is not much wastage generated, making it an environmentally friendly approach. It is at a mature stage in development making it suitable for a varied range of applications as well. The design to production time is reduced, and the direct deposition tool minimizes the material wastage. Additive manufacturing techniques are adopted to fabricate the tactile sensors with tailored geometries to suffice the dimensional requirement and to improve their performance. The limitations of this technology are the requirement of suitable materials. The ink follows specific rheological conditions with a narrow range. Moreover, the addition of surfactants or binders required to stabilize the ink can have a negative effect on the sensing performance of the printed device. Uneven drying or curing of the printed surface can lead to coffee-ring patterns in case of low viscosity inks [2]. Overall, additive manufacturing techniques implemented to develop flexible/stretchable tactile sensors have paved the way in compliant sensing systems conforming to unstructured surfaces. Further requirements of novel printable materials call for manageable and cheap manufacturing with renewable supplies. As the world moves towards smart and intelligent systems in every aspect, it is envisioned that in the future, the incorporation of the various stretchable device mechanisms in the 3D printing fabrication methods will enable a wider application range.

## Figures and Tables

**Table 1:** Features of the additive manufacturing techniques frequently used for the fabrication of flexible/stretchable tactile sensors.

Fused filament fabrication (FFF) [18, 233]	Aerosol jet printing	Inkjet printing [33]	Direct ink writing (DIW)	Electrohydrodynamic printing (E-jet) [44, 234, 235]
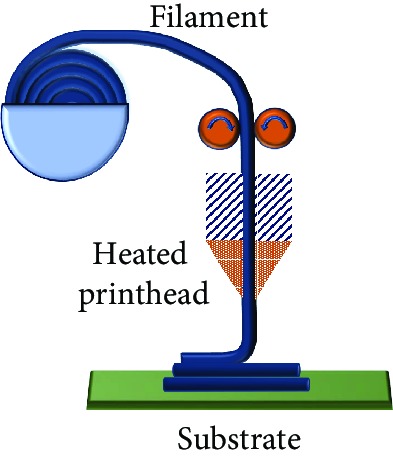	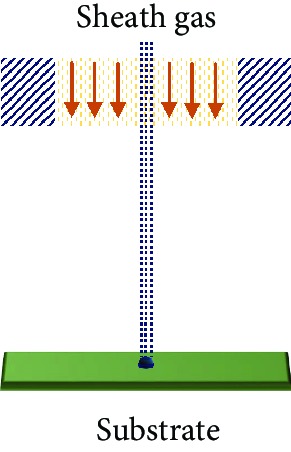	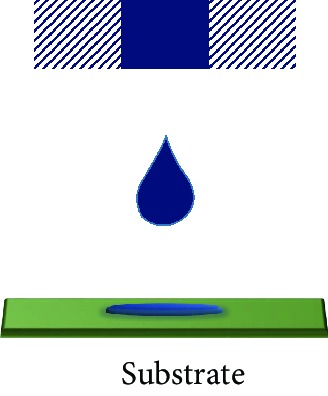	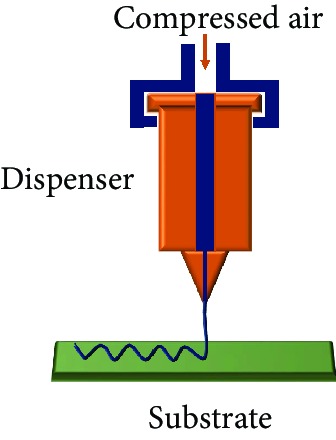	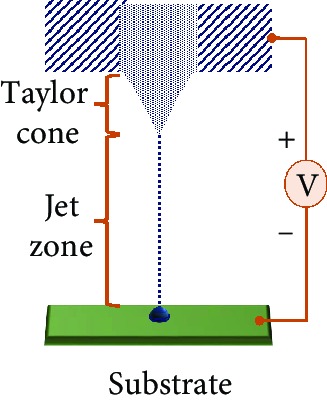

*Technology*				
Heated nozzle by thermal energy	Aerodynamic focussing	Thermal or piezoelectric	Pneumatic or syringe nozzle	Driven by electric field

*Flexibility of printed element*				
★★★☆☆	★★★★☆	★★★★☆	★★★★★	★★★★★

*Stretchability of printed element*				
★★☆☆☆	★★★★☆	★★★☆☆	★★★★★	★★★★★

*Flexible/stretchable element (FSE) material*				
Thermoplastic, composites made into filaments	Conductive inks, dielectrics of viscosity (1–1000 cP)	Any low viscosity inks (1–20 cP)	Any type of flowable ink	Polymer, nanoparticle inks

*Dimensional accuracy*				
50–500 *μ*m	10–250 *μ*m	20-100 *μ*m	250 nm–100 *μ*m	Few hundred nm-*μ*m

*Advantages*				
(i) Inexpensive machine and materials (ii) Possibility of multimaterial printing (iii) Adjustable temperature of nozzle and build platform (iv) Mild usage and maintenance	(i) Printability complex nonplanar surfaces (ii) High resolution (iii) Clog-resistant nozzle (iv) Continuous stream (v) Highly focussed (vi) Low processing temperature	(i) Cost-effective (ii) High throughput (iii) Wide range of materials (iv) Low material wastage (v) Drop-wise material deposition	(i) Highly versatile (ii) Wide range of materials (iii) Possibility of multimaterial printing	(i) Noncontact printing (ii) High resolution (iii) Broad range of materials (iv) Multimode printing (v) Ability to control jet emission

*Disadvantages*				
(i) Nozzle too close to the substrate (ii) Heating effect may damage the printed trace (iii) Rough surface finish of printed pattern (iv) Unable to build sharp features (v) Limited dimensional accuracy (vi) Materials need to be made into filaments	(i) Droplet carrier creates a cloud of powder at the printed spot (ii) Sheath gas inhibits the local bonding of printed trace by solidifying or crystallizing it locally	(i) Nozzle clogging (ii) Slow speed compared to the rest (iii) High contact angle produces bulging of printed traces (iv) Potential coffee-ring effect due to unequal distribution (v) Random directionality of drops	(i) Nozzle too close to the substrate (ii) Might require postprocessing for some materials (iii) Challenge to maintain mechanical integrity and shape during printing	(i) High electrical forces during ejection cause printed feature to be much smaller than nozzle diameter (ii) Insulating materials affect the intensity of the electric field (iii) Low maximum height < 5 mm

**Table 2:** Various types of printing methods used for the fabrication of flexible/stretchable tactile sensors.

Year	Fabrication technique	Printer used	Minimum detail	Printed component	Transduction principle	Ref.
2019	Fused filament fabrication (FFF)	Customized FFF printer	—	Sensing element	Piezoresistive	[236]
2019	Fused filament fabrication (FFF)	MakerBot 2X replicator	200 *μ*m	Sensing element, sensor body	Piezoresistive	[237]
2019	Fused filament fabrication (FFF)	Customized LulzBot	—	Sensing element	Capacitive	[181]
2019	Aerosol jet printing	Self-built printer	~10 *μ*m	Sensing element	Piezoresistive	[238]
2019	Direct ink writing	Self-built printer	—	Sensing element	Piezoresistive	[239]

2018	Fused filament fabrication (FFF)	Standard FFF printer	—	Sensor body, sensing element	Piezoresistive	[130]
2018	Inkjet printing	Enjet Corp., Korea	60 *μ*m	Sensor electrode	Piezoresistive	[240]
2018	Inkjet printing	Canon IP100	—	Sensing element	Capacitive	[213]
2018	Direct ink writing	Self-built printer	60 *μ*m	Sensing element	Piezoresistive	[149]
2018	Direct ink writing	Glass micropipette	—	Sensor electrodes	Capacitive	[82]
2018	Direct ink writing	Home-built 3D printer, Cura Ultimaker	400 *μ*m	Sensing element	Capacitive	[191]
2018	Direct ink writing	Custom-built printer	—	Whole sensor	Piezoresistive	[212]
2018	Direct ink writing	Self-built printer	300 *μ*m	Sensing element	Piezoresistive	[241]
2018	Direct ink writing	Home-built 3D printer	400 *μ*m	Sensing element	Capacitive	[242]

2017	Fused filament fabrication (FFF)	MakerBot 2X replicator	100-300 *μ*m	Sensor body, sensing element	Piezoresistive	[243]
2017	Fused filament fabrication (FFF)	MakerGear M2	50 *μ*m–0.25 mm	Sensing element	Piezoresistive	[244]
2017	Inkjet printing	ProJet 5500X	~30 *μ*m	Sensor body	Piezoresistive	[9]
2017	Inkjet printing	—	—	Sensor electrode	Piezoresistive	[245]
2017	Inkjet printing	MicroFab jetlab II	—	Dielectric layer	Capacitive	[198]
2017	Inkjet printing	jetlab4, MicroFab	170 nm	Sensor element	Piezoelectric	[246]
2017	Direct ink writing	Custom-built 3D printer	—	Sensor body, sensing element	Piezoresistive	[231]
2017	Direct ink writing	Self-built	—	Sensing element	Piezoresistive	[247]
2017	Direct ink writing	Self-built printer	Trace width—100 *μ*m	Sensing element and electrodes	Capacitive	[109]
2017	Electrohydrodynamic (EHD)	Self-built printer	15 *μ*m	Sensing element	Capacitive	[235]

**Table 3:** 3D printing of tactile sensors with their fabricated materials and sensor features.

Year	Transduction principle	Feature	Measured constrain	Active material	Substrate material	Electrode/interconnects material	Sensitivity	Gauge factor	Measured range	Cyclic stability	Ref.
2019	Piezoresistive	Flexible	Strain	TPU/graphene	—	—	—	80	Up to 200%	6000 strain cycles	[236]
2019	Piezoresistive	Bidirectional	Strain	TPU/MWCNT	TPU	—	—	1.5-3	Up to 50%	20 cycles	[237]
2019	Piezoresistive	Stretchable	Strain	AgNP/MWCNT	Ecoflex 0030	Ag conductive paste	—	58.7	Max strain limit 74%	1000 strain cycles	[238]
2019	Piezoresistive	Fast response 171 ms, thickness 650 *μ*m	Pressure	Graphene nanoplatelets/MWCNT/polyethylene oxide (PEO)	PDMS	Ag conductive paste	6.56 MPa^−1^ for <65 kPa 0.335 kPa^−1^ for 100 kPa	—	14–105 kPa	1000 pressure cycles	[239]
2019	Capacitive	Bimodal sensor	Bending strain, pressure	Ag conductive ink	Polyethylene Naphthalate (PEN), paper	—	—	3500	0.41–1.10% strain	4500 bending cycles	[35]
2019	Capacitive	Stretchable	Strain	Carbon black/Ecoflex	Barium titanate/Ecoflex	—	—	1.7 and 0.3	—	1000 stretching cycles	[181]

2018	Piezoresistive	High sensitivity	Bending strain, pressure	Polylactic acid-graphene (PLA-G) filament	Thermoplastic polyurethane filament (TPU)	—	—	~550	292 Pa–487 kPa 0.1–26.3 degrees	—	[130]
2018	Piezoresistive		Pressure	PVDF-HFP/PEDOT:PSS	PET	PEDOT:PSS	13.5/kPa	—	—	10000 strain cycles	[240]
2018	Piezoresistive	Meniscus-guided printing mechanism	Strain	MWCNT/polyvinylpyrrolidone (PVP)	Polyimide (PI), polymethyl methacrylate (PMMA)	Ag paste, copper wire	—	13.07 and 12.87	—	~1500 bending cycles	[149]
2018	Piezoresistive	Stretchable	Pressure	Thermoplastic polyurethane (TPU)/carbon black/NaCl	PDMS	Ag microflake/TPU composite	5.54 kPa^−1^, 0.123 kPa^−1^, 0.0048 kPa^−1^	—	10 Pa to 800 kPa	10000 pressure cycles	[212]
2018	Piezoresistive	Flexible	Pressure	MWCNT/TangoPlus	TangoPlus	—	∼0.5 N	—	0–50 N	—	[241]
2018	Capacitive	Tactile perception	Force	Ag nanoparticle ink	PET	—	—	—	—	—	[213]
2018	Capacitive	Stretchable	Pressure	Ecoflex	PDMS	AgNW ink	10.6%/kPa	—	100 Pa–6 kPa	2000 stretching cycles	[82]
2018	Capacitive	Stretchable	Strain	CNT/PDMS	PDMS	AgNW solution	—	0.95–0.77	—	—	[191]
2018	Capacitive	Stretchable	Pressure	CNT/PDMS	PDMS	—	547.9 kPa^−1^	—	0–110 Pa	500 pressure cycles	[242]
2018	Triboelectric	Flexible	Compressive load	Acrylonitrile butadiene styrene (ABS)	—	Ionic hydrogel, copper wire	—	—	—	3000 working cycle	[202]

2017	Piezoresistive	Bendable	Strain	Ag nanoparticle ink	VisiJet composite photopolymer	—	—	50	0-10% strain	—	[9]
2017	Piezoresistive	Flexible multiaxial force detection	Force	CNT/thermoplastic polyurethane nanocomposite filament	Thermoplastic polyurethane filament (TPU)	Ag paste	—	—	0-5 N	1000 bending cycles	[243]
2017	Piezoresistive	Flexible bimodal	Pressure	PEDOT:PSS/polyurethane dispersion (PUD)	PDMS	Ag nanoparticle	3 Pa	—	3 Pa to 5 kPa	100000 pressure cycles	[245]
2017	Piezoresistive	Stretchable	Pressure	Ag/silicone	Silicone ink, Dragon Skin 10	Ag/silicone	—	~180	—	100 pressing cycles	[231]
2017	Piezoresistive	Stretchable	Strain	NH_2_-MWCNT/GO/SIS	Polystyrene-polyisoprene-polystyrene (SIS)	Cu wires, Ag epoxy	—	>70	—	—	[247]
2017	Piezoresistive	Stretchable	Strain	Ag nanoparticles	Poly(styrene-b-butadiene-b-styrene) SBS	—	—	—	—	300 stretching cycles	[248]
2017	Capacitive	Hybrid 3D printing	Strain, pressure	Ag flakes	Pure TPU	Ag flakes in thermoplastic polyurethane (TPU) ink	—	≈13.3	0–3 MPa	1000	[109]
2017	Capacitive	Flexible	Touch force	Ag nanoparticle ink	Glass, PET film	—	—	—	—	—	[235]
2017	Capacitive	Very high sensitivity	Pressure	n-Butyl acetate diluted PDMS	ITO-coated glass	—	10.4 kPa^−1^	—	0-70 Pa	—	[198]
2017	Piezoelectric	Energy generation	Pressure	P(VDF–TrFE)	Polyethylene Naphthalate	Silver nanoparticle	—	—	—	—	[246]

**Table 4:** Properties of a few commonly used substrate materials [118, 138, 148, 249–251]. ^∗^Subject to change with fabrication.

Material	Thickness (*μ*m)	Density (g/cm^3^)	Transparency (%)	Tg (°C)	Max. allowable temp (°C)	Thermal coeff. (ppm/°C)	Young's modulus (GPa)	Tensile strength (MPa)	Solvent resistance	Surface energy (dyn/cm)
PET	16-100	1.39	90	70-110	150	15-33	2-4.1	55-250	Good	35-50
PEN	12-250	1.36	87	120-155	260	20	0.1-0.5	280-550	Good	20–35
PI	12-125	1.36–1.43	35-60	155-270	Up to 400	8-20	2.5-3	85-300	Good	40
TPU	—	1.18	90	80	130	153	7 MPa	28-54	Good	
Paper (transparent nanofiber)	20-200	1.53	80	200	150	—	13	223	Poor	50-60
Ecoflex	^∗^	1.07	70-90	—	232	284	0.05–0.10 MPa	0.83–2.41	Poor	35
PDMS	^∗^	0.965	90	-125	200	270-310	1.84 MPa	2.24	Poor	10-20

**Table 5:** Properties of a few commonly used active materials [79, 252, 253].

Materials		Electrical conductivity	Thickness (nm)	Length (*μ*m)	Transparency (%)	Young's modulus (GPa)	Sheet resistance (*Ω*/sq)
Ag	Nanoparticle	6.2 × 10^5^ S/cm	20-150 (nanowire)	1-50 (nanowire)	85–95	83	10–70
	Nanowire	1.6 × 10^‐6^ *Ω* cm					

Cu	Nanoparticle	5.9 × 10^5^ S/cm	5-120 (nanowire)	10-50 (nanowire)	85–95	130	10–70
	Nanowire	1.740 × 10^‐6^ *Ω* cm					

Ni	Nanoparticle	1.4 × 10^5^ S/cm	10-200 (nanowire)	1-5000 (nanowire)	85–95	—	10–70
	Nanowire	30–50 × 10^−6^ *Ω* cm					

CNT	SWCNT	~10^4^ S/cm	0.4-50	1-1000000	70–90	~1000	60–485
	DWCNT	~10^4^ S/cm				—	
	MWCNT	~10^4^ S/cm				300–1000	

PEDOT:PSS film		~10^3^ S/cm	—	—	Up to 97	0.9–2.8	145-1000

ITO		10^3^-10^4^ S/cm	—	—	Up to 80	116	30-820
